# Low Consciousness in a Patient with Pancolitis

**DOI:** 10.5334/jbsr.3265

**Published:** 2024-02-14

**Authors:** Ani Nikoghosyan, Julie Lambert

**Affiliations:** 1 Department Radiology, UZ Leuven, campus Gasthuisberg, Leuven, Belgium Herestraat 49/B, 3000 Leuven; 2 Department Radiology, UZ Leuven, Campus Gasthuisberg, Leuven, Belgium Herestraat 49/B, 3000 Leuven

**Keywords:** MRI, uremic encephalopathy, lentiform fork sign, metabolic acidosis

## Abstract

*Teaching point:* Lentiform fork sign is a rare finding on MRI of the brain, found in patients with metabolic acidosis, in a critically ill patient with low consciousness and renal failure, this sign can correspond to uremic encephalopathy.

## Case History

An 18-year-old male with a known medical history of ulcerative colitis was referred to the intensive care unit (ICU) of the University Hospitals of Leuven due to an exacerbation of a severe ulcerative pancolitis. There was no improvement despite treatment with antibiotics in the referring hospital.

**Figure 1:**
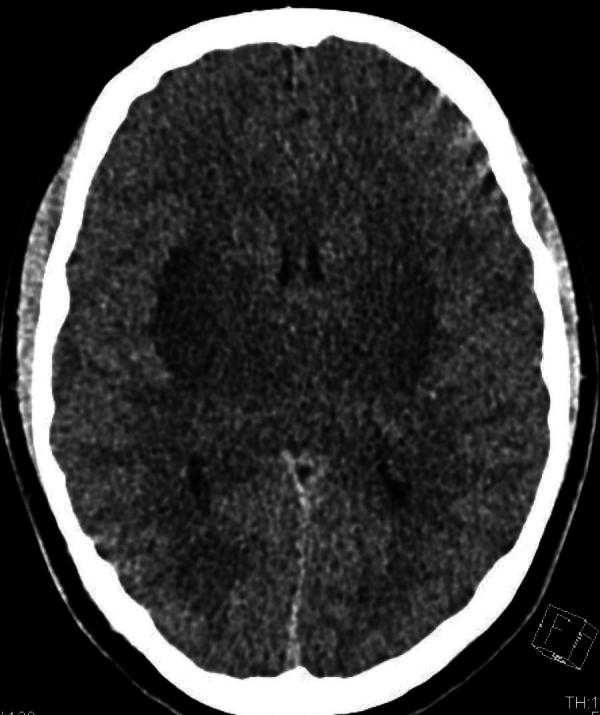
Axial CT scan showing hypodense lentiform nuclei and thalami.

**Figure 2:**
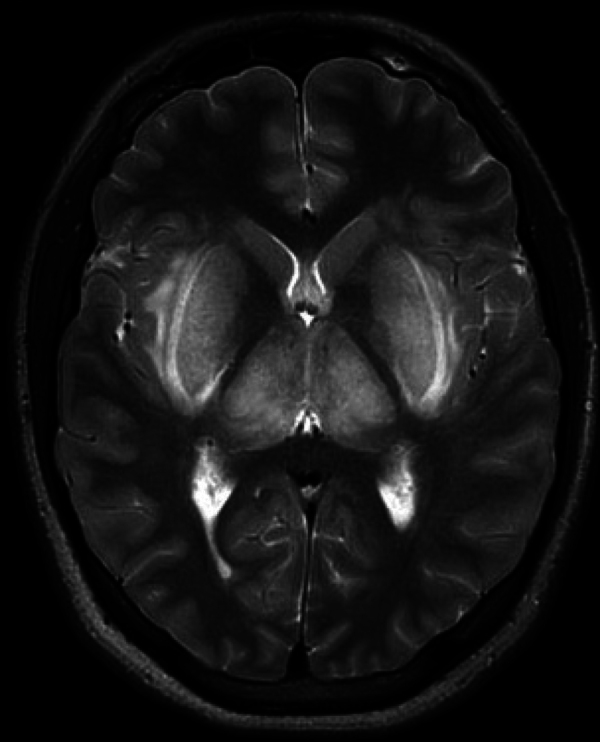
Axial T2 weighted image showing hyperintensity in the lentiform nuclei and in the thalami.

**Figure 3:**
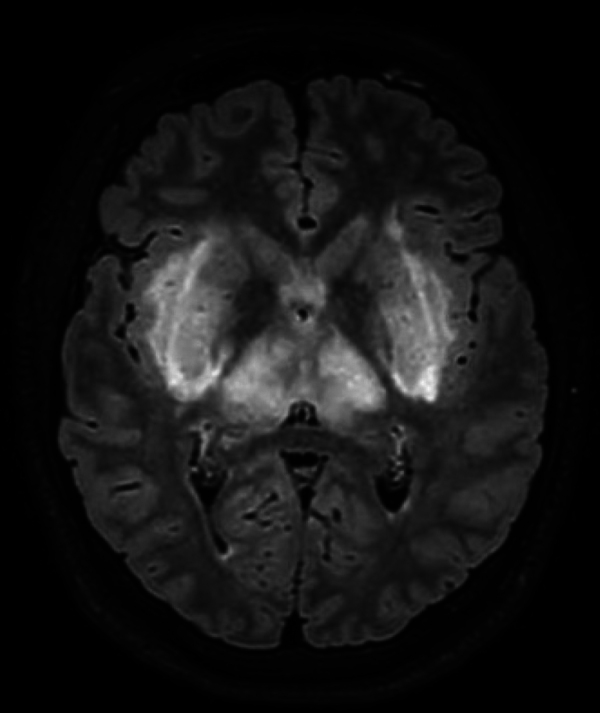
Axial FLAIR image showing hyperintensity in the lentiform nuclei and in the thalami.

**Figure 4:**
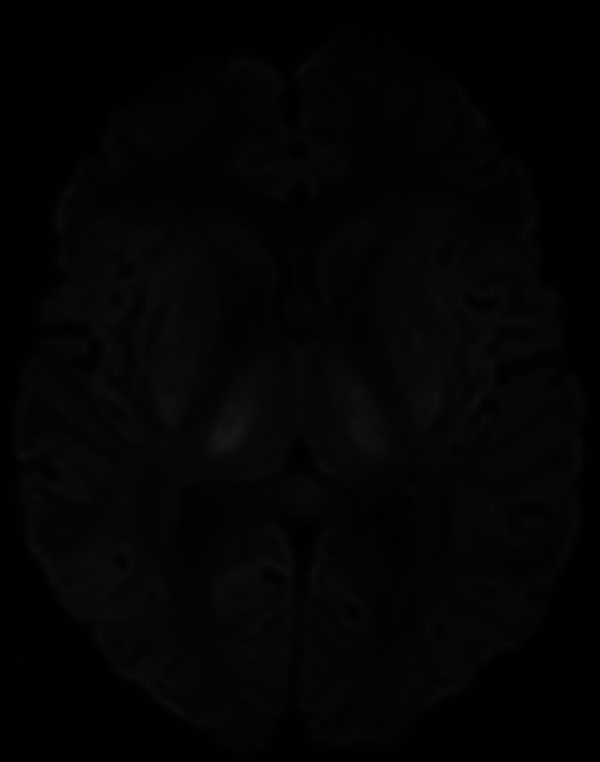
Axial B1000 DWI image showing slightly higher signal in the lentiform nuclei and laterally in the thalami.

The patient had developed acute renal failure. On the ICU, he had a slightly distended abdomen, low consciousness, and inadequate behavior. The patient had received a CT scan of the brain on the same day. On this CT, narrowed sulci and slit-like lateral ventricles were seen and both the external capsule and lentiform nucleus were abnormally hypodense, suggestive for edema (Figure 1). Additionally, an MRI of the brain was performed, which showed a symmetrical, linear T2, and FLAIR hyperintense signal in the external capsule and in the lentiform nucleus bilaterally, compiling the lentiform fork sign, together with slightly increased signal intensity in the thalami (Figures 2 and 3). There was diffusion restriction in the same central regions of the brain (Figure 4).

The patient had undergone a total colectomy and received kidney dialysis with appropriate supportive therapy. After 1 month, on follow-up MRI of the brain, there was regression of earlier described abnormalities and the patient recovered.

## Comments

The lentiform fork sign represents a rare neuroradiological finding on MRI, comprised by the combination of symmetrically increased T2/FLAIR signal intensity in the lentiform nuclei and brightly hyperintense rim in the internal and external capsule, surrounding both putamina and thus, resembling a fork. This sign is seen in patients with acute metabolic acidosis, possibly by uremic encephalopathy, but also in hypoglycemic encephalopathy and in methanol intoxication.

There are three patterns of presentation of uremic encephalopathy: the first is involvement of the basal ganglia with the lentiform fork sign, the second is cortical or subcortical involvement, and the last pattern is the white matter involvement [1]. The most common pattern of uremic encephalopathy is the central type with involvement of the lentiform nuclei and in the basal ganglia in general. We have to differentiate the central type of uremic encephalopathy from other pathologies, such as methanol intoxication, hepatic encephalopathy, hypoxic-ischemic encephalopathy, and Wilson disease. In encephalopathies, due to metabolic acidosis, the involvement of basal ganglia is common and differentiation is not always obvious. Therefore, clinical findings may help to make the further differentiation.

In conclusion, the lentiform fork sign is a characteristic finding in the clinical context of possible metabolic acidosis, as in uremic encephalopathy.
